# A Contemporary Review of Resectability and Treatment Strategies for Colorectal Liver Metastases: Part 2—Advanced Surgical Techniques, Conversion Strategies, and the Expanding Boundaries of Treatment

**DOI:** 10.3390/cancers18193169

**Published:** 2026-10-01

**Authors:** Jennifer A. Kalil, René Adam, Nicholas Meti, Peter Metrakos

**Affiliations:** 1 Cancer Research Program, Research Institute of the McGill University Health Center (RI-MUHC), Montreal, QC H4A 3J1, Canada; peter.metrakos@mcgill.ca; 2 Department of Surgery, McGill University Health Center, Montreal, QC H4A 0B1, Canada; 3 Henri-Bismuth Hepatobiliary Center, Paul-Brousse Hospital, Assistance Publique-Hôpitaux de Paris, Paris-Saclay University, 94800 Villejuif, France; rene.adam@aphp.fr; 4 Gerald Bronfman Department of Oncology, McGill University Health Center, Montreal, QC H4A 0B1, Canada; nicholas.meti@mcgill.ca

**Keywords:** colorectal liver metastases, conversion chemotherapy, portal vein embolization, liver venous deprivation (LVD), hepatic artery infusion, future liver remnant, ALPPS, liver-directed therapy

## Abstract

**Background/Objectives**: Most patients with colorectal liver metastases (CRLM) present with disease beyond upfront technical resectability. Advances in systemic therapy, surgical technique, and liver-directed therapies have expanded the proportion of patients who can achieve curative-intent treatment, yet substantial heterogeneity in practice persists. This review provides a structured synthesis of current management strategies for borderline resectable, initially unresectable, and never resectable CRLM. **Methods**: A structured literature search of PubMed/MEDLINE, Embase, Scopus and the Cochrane Library was performed to identify studies published between 2000 and 2026 using the eligibility criteria described in Part 1 of this review. Findings were synthesized narratively across systemic, surgical, and liver-directed treatment strategies. **Results**: Future liver remnant augmentation through regenerative procedures offers higher rates of conversion to resection in borderline cases. Conversion chemotherapy enables secondary resection in 12–35% of initially unresectable patients, with regimen selection guided by RAS/BRAF status, primary tumor sidedness, and performance status; however, most pivotal trials were designed for the broader metastatic colorectal cancer population, with conversion to resection rarely serving as the primary endpoint. For never resectable liver-only disease, liver transplantation demonstrates survival advantage in highly selected patients and iterative local therapy remains an area of active investigation. Across all settings, achieving complete local treatment remains the strongest determinant of long-term survival. **Conclusions**: Management of complex CRLM requires individualized, multidisciplinary decision-making with repeated reassessment of resectability throughout treatment. The evidence is constrained by heterogeneous resectability definitions, reliance on trials not designed for CRLM-specific endpoints, and limited data on several emerging strategies. Standardized eligibility frameworks and CRLM-specific trial designs are needed to advance the field.

## Simple Summary

Most patients with colorectal cancer liver metastases (CRLM) present with unresectable disease. For these patients, treatment strategies have expanded considerably and now include intensified chemotherapy regimens tailored to tumor molecular profile, techniques to grow the remaining liver before surgery, staged operations, liver-directed therapies, and, in highly selected cases, liver transplantation. Despite these advances, much of the evidence guiding treatment decisions comes from trials that were not specifically designed for patients with liver metastases and there is no universal agreement on what constitutes resectability or on perioperative management of systemic therapy. This review summarizes the current evidence for managing patients with borderline resectable, initially unresectable, and never resectable CRLM. It highlights the importance of repeated multidisciplinary reassessment, the emerging role of iterative multimodal treatment strategies, and the need for prospective trials designed specifically for this population.

## 1. Introduction

The boundaries of resectability in colorectal liver metastases (CRLM) have expanded substantially through advances in systemic therapy, liver-directed interventions, and regenerative surgical techniques. In Part 1 of this review, the framework for assessing resectability was established and the management of upfront resectable and synchronous disease was discussed [1]. However, the majority of patients with CRLM present with disease that is not amenable to straightforward resection—whether due to extensive bilobar tumor burden, insufficient future liver remnant (FLR), unfavorable tumor biology, or a combination of these factors [2]. It is in these more complex clinical scenarios that treatment heterogeneity is most pronounced and where evidence-based guidance is most needed.

In borderline resectable disease, neoadjuvant systemic therapy combined with FLR augmentation strategies has enabled curative-intent resection in cases previously considered inoperable [3,4,5]. In the initially unresectable setting, the selection and sequencing of conversion chemotherapy, guided by molecular profiling and primary tumor sidedness, has become increasingly refined, though optimal regimens remain an area of active investigation [6,7,8]. For patients with disease that is never resectable, liver transplantation has emerged as a viable option in highly selected cases, while the role of debulking surgery and liver-directed therapies continues to be defined [9,10,11].

This second part of the review synthesizes the current evidence on the management of borderline resectable, initially unresectable, and never resectable CRLM, with a focus on treatment sequencing, conversion strategies, advanced surgical techniques, and emerging approaches. This work is a narrative review. The literature search followed the structured strategy described in Part 1. PubMed/MEDLINE, Embase, Scopus and the Cochrane Library databases were searched from 2000 to 2026 using terms related CRLM, resectability, conversion therapy, FLR augmentation, and liver-directed therapy. Randomized controlled trials, prospective studies, and meta-analyses were prioritized; retrospective series were included where higher-level evidence was lacking. Reference lists of included articles, clinical practice guidelines, and consensus statements were also reviewed. Study outcomes including overall survival (OS), disease-free survival (DFS), recurrence patterns, and perioperative morbidity are reported and synthesized, and areas of ongoing uncertainty are emphasized.

## 2. Management of Borderline Resectable CRLM

**Figure 1:**
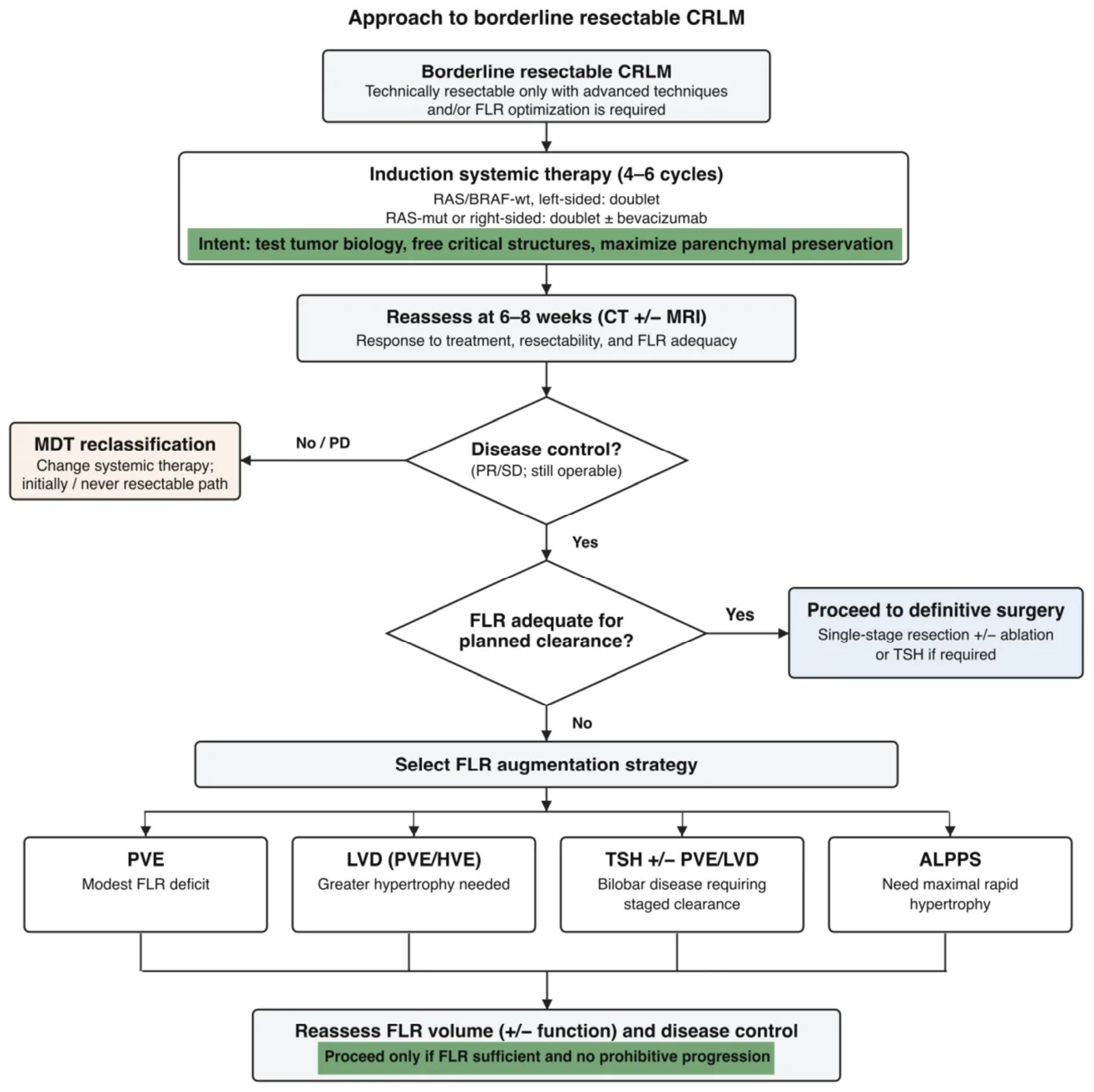
Approach to borderline resectable CRLM. Arrows indicate the sequence of assessment and management within the proposed treatment algorithm. CRLM, colorectal liver metastases; wt, wild-type; mut, mutated; CT, computed tomography; MRI, magnetic resonance imaging; FLR, future liver remnant; MDT, multidisciplinary team; PR, partial response; SD, stable disease; PD, progressive disease; PVE, portal vein embolization; LVD, liver veno-venous deprivation; HVE, hepatic vein embolization; TSH, two-stage hepatectomy; ALPPS, associating liver partition and portal vein ligation for staged hepatectomy.

Borderline resectable CRLM refers to disease burden that is technically resectable at presentation only when advanced surgical techniques are used and the FLR is optimized. Curative resection is feasible, although the operative approach is often more complex and the biology is less favorable [12,13]. Neoadjuvant chemotherapy may be used to improve operability and facilitate maximal parenchymal preservation, but the intent is induction rather than “conversion” from unresectable to resectable disease [14]. In this setting, resection is planned from the outset and systemic therapy is used to test tumor biology and facilitate a safer or parenchymal-sparing operation. By contrast, in initially unresectable disease (Section 3), surgery is contingent on achieving a sufficient chemotherapy response. The management pathway of borderline resectable CRLM is outlined in Figure 1.

### 2.1. Induction Systemic Therapy in Borderline Resectable Disease

Induction therapy is used primarily to shrink metastases, separate lesions from critical structures, and enable a parenchymal-sparing resection. Treatment is typically limited to 4–6 cycles with imaging every 6–8 weeks (approximately every 3–4 biweekly cycles) to assess treatment response. This balances the goal of obtaining sufficient tumor regression against the increased perioperative risk of prolonged chemotherapy exposure [15,16].

#### 2.1.1. Treatment Regimen

Current guidelines recommend fluoropyrimidines (5-fluorouracil, capecitabine) with an oxaliplatin- or irinotecan-based doublet (FOLFOX, CAPOX, FOLFIRI) as the standard first-line backbone, with triplet (FOLFOXIRI) reserved for patients with good performance status and more aggressive tumor biology [17,18]. The addition of anti-epidermal growth factor receptor (anti-EGFR) monoclonal antibody, such as cetuximab or panitumumab, or an anti-vascular endothelial growth factor (anti-VEGF) monoclonal antibody, bevacizumab, is guided by RAS/BRAF status and primary tumor sidedness and is discussed in the context of initially unresectable disease (Section 3), where the intent is genuine downstaging. In the borderline setting, the addition of an anti-EGFR monoclonal antibody is not supported. The New EPOC trial, which enrolled patients with resectable or suboptimally resectable KRAS-wt CRLM, demonstrated shorter median OS with the addition of cetuximab to perioperative chemotherapy (55.4 vs. 81 months; HR 1.45, *p* = 0.036), with the detriment most pronounced in patients with favorable prognostic features [19].

#### 2.1.2. Chemotherapy-Associated Liver Injury (CALI) and Timing to Surgery

Neoadjuvant chemotherapy has been associated with hepatocellular injury, most notably hepatic microvasculature damage resulting in sinusoidal obstruction with perisinusoidal fibrosis and veno-occlusive lesions [20]. The pattern of injury depends on the agent used, with oxaliplatin exposure driving sinusoidal damage, while irinotecan is more often associated with steatohepatitis [20,21].

Evidence is mixed on whether CALI increases operative risk, but most series suggest the hazard is related to the duration of treatment and the interval between the last cycle and surgery [15,22,23]. Review data and pooled analyses indicate a worse short-term prognosis, including higher 90-day mortality, increased major morbidity, and higher incidence of post-hepatectomy liver failure (PHLF) [15,21,24]. However, hepatic resection after neoadjuvant therapy is feasible and safe if timed appropriately [23]. Prolonging preoperative therapy to ≥9 cycles was associated with increased sinusoidal injury and postoperative liver insufficiency without improving pathologic response [15]. Surgical complications decrease when surgery is timed >4 weeks after the last cycle of chemotherapy, dropping from 11% at ≤4 weeks to 5.5% at 5–8 weeks and 2.6% at 9–12 weeks (*p* = 0.009) [23].

### 2.2. Optimization of the FLR

#### 2.2.1. Portal Vein Embolization (PVE)

PVE is performed to stimulate compensatory hypertrophy of the FLR by occluding portal venous blood flow to the liver segments planned for resection, inducing atrophy in the embolized lobe and hypertrophy in the contralateral lobe. Hypertrophy is reported inconsistently across studies. It is reported as the relative increase in FLR volume (percentage hypertrophy), as the absolute change in standardized FLR (degree of hypertrophy, DH), or as the kinetic growth rate (DH per week), and at different time points after the procedure; therefore, values are not directly comparable between series. A systematic review encompassing 1791 patients demonstrated the high technical and clinical success rate of PVE (99.3% and 96.1%, respectively), with a mean relative increase in FLR volume of 38% [3]. Hypertrophy typically plateaus by three weeks, and the mean time to hepatectomy is 37 days. However, 20% of planned liver resections were canceled due to delayed hypertrophy or tumor progression; across series, resection rates after PVE range from approximately 68% to 80% [3,4].

#### 2.2.2. Simultaneous PVE and Hepatic Vein Embolization (HVE)

Simultaneous PVE/HVE combines portal inflow and ipsilateral hepatic venous outflow occlusion to induce rapid FLR hypertrophy. The technique was first described by Guiu et al. in 2016 using a transhepatic approach with vascular plugs and glue for hepatic vein occlusion, termed liver venous deprivation (LVD) [25]. Since then, LVD has evolved from a specific technical descriptor to an umbrella term encompassing simultaneous portal and hepatic vein embolization regardless of access route or embolic agent, and the two terms are used interchangeably in the current literature [26]. The DRAGON 0 cohort, a multicenter retrospective study comparing PVE/HVE with PVE alone in patients with various liver tumors, demonstrated a greater relative increase in FLR volume for PVE/HVE (59% vs. 48%, *p* = 0.020), and a resectability rate of 90% versus 68% (*p* = 0.007), rising to 92% on updated follow-up [4,27]. The DRAGON 1 prospective international multicenter study confirmed these findings, demonstrating a resection rate of 90% among 96 patients with insufficient FLR [28]. The associated morbidity was 19% (17% being Clavien–Dindo Grade I–II) with no procedure-associated mortalities [28,29]. However, the post-hepatectomy 90-day mortality was 8% (7/86 resected patients) and PHLF grade B/C occurred in 22%—rates that likely reflect the complexity of the patient population rather than the embolization technique itself [28]. A pooled meta-analysis of nine comparative studies found LVD reduces the time to hepatectomy by a mean of 6.7 days relative to PVE (95% CI 5.3–8.0 days, *p* < 0.0001), with reported ranges of approximately 30–44 days for LVD and 34–49 days for PVE across individual series [30].

### 2.3. Advanced Surgical Techniques

#### 2.3.1. Two-Stage Hepatectomy (TSH)

TSH involves two sequential liver resections to address bilobar metastatic disease that cannot be safely cleared in a single operation. It distributes surgical risk across stages, allows liver regeneration between procedures, and actively stimulates hypertrophy of the FLR [5]. Additionally, this staged strategy provides an opportunity to assess tumor biology and control disease progression, ensuring that the second stage is performed only when it is both safe and potentially curative [5]. Completion rates range from 60 to 82% across series, with tumor progression being the predominant cause of dropout, followed by insufficient FLR hypertrophy, and postoperative complications [31]. A recent multicenter study confirmed an overall oncologic failure rate of 40% [32]. Despite the attrition, patients who complete both stages achieve 3- and 5-year OS of 60–70% and 32–49%, respectively [31].

#### 2.3.2. Associating Liver Partition and Portal Vein Ligation for Staged Hepatectomy (ALPPS)

ALPPS was designed to address the challenges of dropout rates and tumor progression associated with the 4- to 8-week interstage delay of conventional TSH. This method facilitates rapid and significant hypertrophy of the FLR by combining portal vein ligation with in situ parenchymal transection during the first hepatectomy stage and shortens the interstage interval to 7–12 days [33,34]. Early ALPPS series reported high perioperative mortality of 12% and severe morbidity of 44%, prompting a decade of technical refinements [33,35]. In a systematic review and meta-analysis, ALPPS achieved higher rates of completion hepatectomy and R0 resection compared to conventional TSH, but at the cost of higher major complications (41.7% vs. 17.6%) [34]. A more recent meta-analysis of 5891 patients across 103 studies confirmed that ALPPS achieves the highest mean relative FLR increase (68.5%) and stage 2 feasibility (95%), with morbidity and mortality rates that were not significantly different from PVE/HVE, likely reflecting improvements in technique [36].

#### 2.3.3. Minimally Invasive Approaches and Intraoperative Guidance

Minimally invasive liver resection is now an established approach for CRLM in experienced centers and was endorsed by the Southampton international consensus guidelines [37]. In the OSLO-COMET randomized trial of parenchyma-sparing resection, the laparoscopic approach reduced 30-day postoperative complications compared with open surgery (19% vs. 31%), with similar rates of free resection margins and similar 5-year OS (54% vs. 55%) [38,39]. Laparoscopic and robotic techniques have also been applied to the first stage of TSH and to ALPPS in expert centers, with the potential to reduce adhesions and facilitate the second stage, although this evidence is largely limited to single-center series [40,41]. Because tactile feedback can be reduced during minimally invasive surgery, intraoperative imaging adjuncts are particularly relevant to the disappearing-metastasis problem discussed in Section 3.3. Intraoperative ultrasound (IOUS) remains the standard tool, and near-infrared fluorescence imaging with indocyanine green (ICG) can identify small and superficial lesions. In a prospective study of 40 patients undergoing resection of CRLM, ICG fluorescence detected additional lesions missed by preoperative CT, IOUS, inspection, and palpation in 12.5% of patients [42].

### 2.4. FLR Augmentation—Comparative Hypertrophy and Perioperative Outcomes

Among FLR-augmentation strategies, PVE is the least invasive, with procedure-related major complications occurring in approximately 2.5% of patients and post-hepatectomy major morbidity of 10–30% [3]. LVD carries a similar procedural complication rate (3%) but post-hepatectomy major morbidity of 20–35%, comparable to TSH (16–43%) and ALPPS (20–50%) [27,31,34,43,44].

Hypertrophy performance differs across techniques. In the Lüthy et al. meta-analysis, LVD achieved a mean relative FLR increase of 36.8%—significantly greater than PVE alone (mean difference 24.5%, *p* < 0.01). However, stage 2 feasibility after LVD was similar to that after PVE. ALPPS achieved the highest hypertrophy of all techniques (discussed in Section 2.3.2) [36]. In the Bozkurt et al. network meta-analysis, LVD achieved the greatest absolute FLR increase among percutaneous techniques and was associated with significantly lower rates of PHLF compared with both PVE (OR 0.35) and ALPPS (OR 0.28) [45]. ALPPS achieves the most rapid hypertrophy and the highest stage two completion rates (90–95% across meta-analyses), but volumetric gains may overestimate functional recovery [36,46]. After stage one, median liver volume increased by roughly 78% while functional gain on hepatobiliary scintigraphy was approximately 29%, with no significant correlation between volume and function [47,48].

In the only randomized comparison of TSH and ALPPS (LIGRO Trial), completion to hepatectomy was 92% with ALPPS versus 57% with TSH (*p* < 0.0001), while major morbidity and 90-day mortality were similar in each group [44]. TSH remains limited by interstage dropout rates of 18–43%, driven primarily by tumor progression and insufficient hypertrophy [32,44]. The most commonly reported dropout rate is approximately 25–35%, although individual series show considerable variability, ranging from 18% in a large multicenter LiverMetSurvey analysis to 43% in the randomized LIGRO trial, reflecting differences in patient selection, chemotherapy protocols, and reassessment timing across centers [31,44,49].

**Figure 2:**
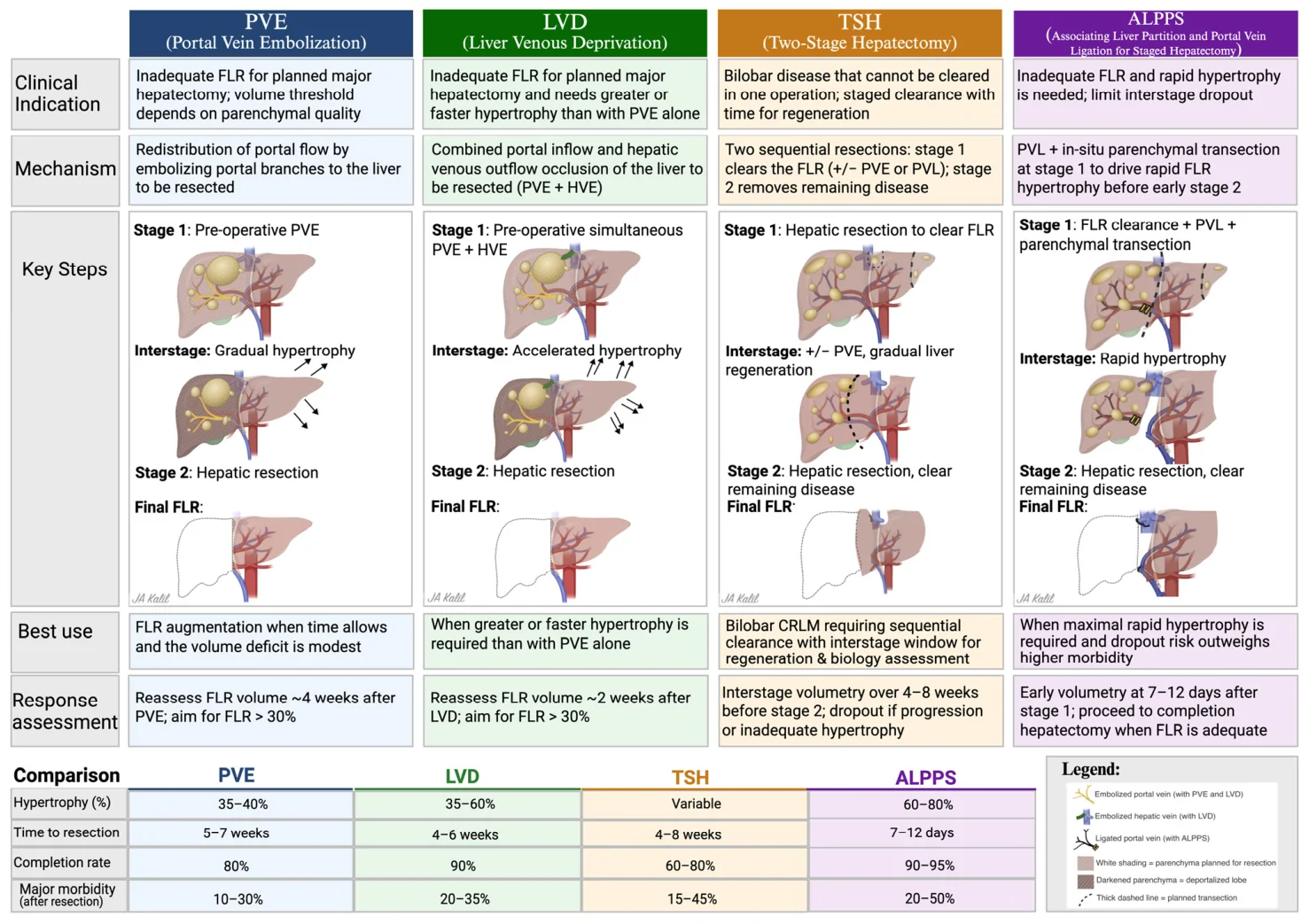
Comparison of strategies for future liver remnant augmentation prior to major hepatectomy. The figure compares portal vein embolization (PVE), liver venous deprivation (LVD), two-stage hepatectomy (TSH), and associating liver partition and portal vein ligation for staged hepatectomy (ALPPS), highlighting their indications, mechanisms, key procedural steps, expected hypertrophy, timing to resection, and clinical outcomes. Values are approximate ranges across heterogenous series with differing definitions of hypertrophy; sources are cited in Section 2.2, Section 2.3, Section 2.4 and Section 2.5. Arrows shown in the interstage step indicate hypertrophy of the FLR. FLR, future liver remnant; HVE, hepatic vein embolization; PVL, portal vein ligation; CRLM, colorectal liver metastases.

Technique selection should be tailored according to tumor burden distribution, baseline FLR deficit, urgency of resection, and institutional expertise (Figure 2). PVE remains appropriate when the deficit is modest. LVD offers enhanced hypertrophy with lower PHLF risk and percutaneous feasibility. ALPPS should be reserved for cases requiring maximal rapid hypertrophy where the risk of interstage dropout outweighs the higher procedural morbidity.

### 2.5. FLR Augmentation—Long-Term Oncologic Outcomes

Despite differences in hypertrophy kinetics and completion rates, long-term oncologic outcomes are largely comparable across techniques. In the DRAGON 0 cohort, R0 resection and recurrence were similar between PVE/HVE and PVE alone, while 3-year OS favored PVE/HVE (median not reached vs. 20 months, *p* = 0.004; multivariable HR 0.57, 95% CI 0.33–0.99), including the CRLM subgroup (median not reached vs. 26 months, *p* = 0.036); however, this survival advantage has not been consistently observed in other studies [4,27,50]. In a comparative series of LVD and ALPPS, the R0 resection rates were similar (ALPPS 94% vs. LVD 100%, *p* = 0.27), but intrahepatic recurrence was higher after ALPPS (100% vs. 52.9%, *p* = 0.002), with 3-year OS of 54.7% after LVD vs. 27.4% after ALPPS, a difference that was not significant (*p* = 0.10) in this series of 17 LVD and 15 ALPPS patients [51]. Given the small sample and retrospective design, this finding should be interpreted with caution. In the largest CRLM-specific comparison of PVE/HVE and ALPPS (n = 214, 8 centers), median OS was 42 vs. 30 months and median DFS was 7 vs. 6 months, with neither difference reaching significance, though resectability on intention-to-treat analysis was higher with ALPPS (98.9% vs. 84.3%) [52]. Comparisons of ALPPS with conventional TSH similarly demonstrate higher completion and R0 resection rates with ALPPS, but equivalent survival outcomes. The LIGRO trial similarly found superior resection rates with ALPPS, and pooled analyses found no survival advantage for ALPPS over TSH [34,44,46]. Pooled meta-analytic data across 1263 patients confirmed that while ALPPS achieves the fastest hypertrophy and highest completion rates, it was associated with worse 1-year DFS compared to PVE (OR 0.45, *p* = 0.03), with all other survival comparisons failing to reach significance [53].

LVD achieves oncologic outcomes comparable to ALPPS, although the lower recurrence rate after LVD derives from small series, while ALPPS improves technical radicality over TSH without a clear survival gain. Selection of surgical strategy should therefore prioritize reliable R0 clearance while recognizing that long-term outcomes remain strongly influenced by tumor biology and the burden of disease rather than by the augmentation method alone.

## 3. Management of Initially Unresectable Disease

Initially unresectable CRLM refers to disease that cannot be completely cleared while preserving adequate FLR and vascular/biliary integrity, even with advanced surgical techniques, because disease distribution is too extensive. Historically, these patients were treated with systemic chemotherapy alone [54]. Approximately 80% of patients with CRLM present with disease beyond technical resectability, but advances in systemic therapy and surgical technique have shifted the paradigm toward pursuing tumor regression to enable resection with curative intent [2].

Optimal management of initially unresectable disease requires multidisciplinary team (MDT) evaluation to distinguish between patients who are potentially convertible to resectability and those who are unlikely to achieve operability [17]. The primary objective of chemotherapy in this setting is to induce sufficient tumor downsizing to enable surgical resection [54,55]. Approximately 12% to 35% of patients can be converted to resectability, with some series reporting rates as high as 55% to 75%, particularly among patients with less biologically aggressive disease [6,55,56,57,58,59,60]. This wide range reflects differences in how unresectability is defined, in patient selection, in whether combined resection-ablation is counted as conversion, and in the denominator used (all treated patients versus trial-eligible patients). The choice and intensity of systemic therapy are central to achieving conversion, with regimen selection guided by disease extent, mutational profile, and patient performance status.

### 3.1. Systemic Therapy for Conversion

Several clinical trials have investigated systemic therapy regimens for patients with initially unresectable metastatic colorectal cancer (mCRC), aiming to achieve tumor shrinkage and facilitate secondary resection. Although most were not exclusively designed for liver-limited disease, their findings have informed and shaped current strategies for managing CRLM.

Among cytotoxic backbones, irinotecan- and oxaliplatin-based doublets have long served as the foundation for conversion therapy, with triplet regimens introduced to maximize tumor response in appropriately selected patients.

#### 3.1.1. Performance Status

Patient performance status represents a fundamental determinant of the intensity and feasibility of systemic therapy in patients with initially unresectable CRLM. Patients with excellent performance status are most likely to tolerate intensified chemotherapy regimens, which offer increased objective response rates (ORR) and progression-free survival (PFS) in appropriately selected patients [61]. Beyond chemotherapy tolerance, a meta-analysis of 13 first-line chemotherapy trials in mCRC identified performance status as an independent and robust prognostic factor for OS [62]. Similarly, retrospective cohort studies have suggested worse performance status is independently associated with worse OS [63].

#### 3.1.2. Doublet and Triplet Backbones

Early studies established the efficacy of irinotecan- or oxaliplatin-based doublets and are widely used in the conversion setting due to their favorable efficacy-to-toxicity profile. In a prospective study by Pozzo et al., FOLFIRI enabled 32.5% of patients with initially unresectable liver metastases to undergo secondary resection, with a median time to progression of 14.3 months and all resected patients alive at a median follow-up of 19 months [54]. In the NCCTG phase II trial, FOLFOX achieved tumor reduction in 60% of patients and conversion to resection in 40% after a median of six months of therapy [64]. Pooled analyses of 8 randomized controlled trials (RCTs) comparing CAPOX and FOLFOX found that the two regimens demonstrated similar PFS, OS, and response rates [65]. While these regimens exhibit comparable efficacy in management of CRLM, each treatment is associated with distinct safety profiles that may influence their suitability for individual patients (Table 1).

Intensified regimens incorporating triplet chemotherapy, such as FOLFOXIRI, are reserved for patients with excellent performance status who can tolerate greater toxicity. These combinations have demonstrated higher response rates compared with doublet regimens in fit patients and are particularly suitable for those with high tumor burden that is liver-dominant [66]. In a phase III trial evaluating FOLFOXIRI and FOLFIRI as first-line agents in treatment of mCRC, FOLFOXIRI achieved superior R0 resection rates (15% vs. 6%, *p* = 0.033) and longer median OS (22.6 vs. 16.7 months, *p* = 0.032) compared with FOLFIRI [61]. The HORG trial, using a similar design, likewise showed higher rates of R0 resection with FOLFOXIRI (R0 10% vs. 4%, *p* = 0.08) without a significant survival difference [67]. These findings have been confirmed in several subsequent studies showing that FOLFOXIRI, particularly when combined with bevacizumab, yields higher overall and complete response rates [68].

#### 3.1.3. RAS and BRAF Status

The integration of biologic agents with chemotherapy has transformed the management of initially unresectable CRLM. Biologic agent selection is guided by molecular profile and is central to optimizing conversion potential and is summarized in Table 2.

In patients with RAS wild-type disease, anti-EGFR agents achieve greater tumor shrinkage and depth of response compared to bevacizumab. The FIRE-3 trial demonstrated superior OS with cetuximab plus FOLFIRI over bevacizumab plus FOLFIRI, and PEAK showed a similar but non-significant OS trend with panitumumab plus mFOLFOX6; together with PARADIGM, these trials established anti-EGFR therapy as preferred in this population [7,8,69,70]. The CELIM trial further supported this approach in the conversion-specific setting, showing that cetuximab combined with either FOLFOX or FOLFIRI increased resectability from 32% to 60% in KRAS wild-type disease [72]. Intensification with triplet chemotherapy has also been an area of investigation. The VOLFI trial demonstrated higher resection rates with the addition of panitumumab to FOLFOXIRI versus FOLFOXIRI alone (33.3% vs. 12.1%, *p* = 0.02), while the TRIPLETE trial found no PFS advantage with triplet over doublet chemotherapy combined with panitumumab, although the final analysis showed longer OS with the triplet (41.1 vs. 33.3 months; HR 0.79) [73,74,75].

For RAS-mutated disease, anti-EGFR therapies are contraindicated due to lack of efficacy and possible detrimental effects [82,83,84]. The BECOME trial, limited to RAS-mutant CRLM, demonstrated higher R0 resection rates with FOLFOX plus bevacizumab compared with FOLFOX alone (22.3% vs. 5.8%, *p* < 0.01) [76]. Triplet chemotherapy plus bevacizumab further improves outcomes in fit patients. The TRIBE and TRIBE2 trials showed improved ORR, PFS and OS with FOLFOXIRI plus bevacizumab compared to FOLFOX or FOLFIRI plus bevacizumab [68,77]. Similarly, the OLIVIA study, restricted to CRLM, confirmed superior R0 rates (49% vs. 23%) and overall resection rates (61% vs. 49%) with FOLFOXIRI plus bevacizumab [59].

BRAF V600E-mutated mCRC, historically associated with dismal prognosis, has undergone a paradigm shift [85]. Earlier data, including subgroup analyses from the CALGB/SWOG 80405 trial, supported the use of chemotherapy plus bevacizumab as the standard first-line approach in this molecular subgroup [86]. However, the phase III BREAKWATER trial has since established encorafenib plus cetuximab combined with chemotherapy as the new first-line standard, demonstrating median OS of 30.3 versus 15.1 months with standard care (HR 0.49, *p* < 0.001) and median PFS of 12.8 versus 7.1 months (HR 0.53, *p* < 0.001)—bringing survival in BRAF-mutant disease closer to that of BRAF wild-type patients [79,80].

#### 3.1.4. MSI-H/dMMR or POLE/POLD1 Mutation-Positive Tumors

Microsatellite instability–high (MSI-H) or mismatch repair–deficient (dMMR) colorectal cancers represent a biologically distinct subset, which comprises less than 10% of CRC, characterized by high tumor mutational burden and increased immunogenicity [87]. Immune checkpoint inhibitors have demonstrated superior efficacy compared with cytotoxic chemotherapy and have become the preferred first-line option for this subgroup of patients with unresectable disease. The KEYNOTE-177 trial established pembrolizumab as the standard of care, showing a significant PFS advantage over chemotherapy (16.5 vs. 8.2 months; HR 0.60; *p* = 0.0002) with improved tolerability [81].

Similarly, the CheckMate 142 study demonstrated durable responses with first-line nivolumab plus low-dose ipilimumab, achieving an objective response rate of 69%, a complete response rate of 13% and disease control in 84% of patients [88]. Although evidence specific to conversion resection remains limited, dramatic tumor regressions have been reported following immunotherapy in MSI-H or POLE/POLD1-mutated disease [81,88].

#### 3.1.5. Primary Tumor Sidedness

Primary tumor sidedness is an independent prognostic and predictive factor that further refines biologic agent selection. Right-sided tumors are associated with shorter survival compared to left-sided tumors and are enriched for KRAS, BRAF, PIK3CA, and SMAD4 mutations, likely explaining the differential sensitivity to anti-EGFR therapy [89].

Sidedness-stratified analyses from the CALGB/SWOG 80405 and PARADIGM trial demonstrate that anti-EGFR therapy provided maximal benefit in RAS wild-type, left-sided disease, while bevacizumab-based regimens are preferred for right-sided primaries regardless of RAS status [8,90].

The CAIRO5 trial further refined biologic and chemotherapy selection according to molecular profile and sidedness in patients with RAS/BRAF-mutated and/or right-sided initially unresectable CRLM [6]. Although FOLFOXIRI plus bevacizumab achieved higher conversion rates than doublet plus bevacizumab, this did not translate into an OS difference [78]. Notably, patients who underwent complete local treatment in either arm had markedly improved OS compared with those who did not, suggesting that the ability to achieve resection remains the dominant determinant of long-term outcomes [6].

Collectively, these data underscore that biologic selection should integrate both molecular and anatomic determinants. Anti-EGFR therapy offers the greatest benefit in RAS wild-type, left-sided tumors, while bevacizumab-based regimens remain preferred for RAS-mutant, BRAF-mutant, and right-sided primaries. The choice of chemotherapeutic regimen and biologic is summarized in Table 3.

Although several regimen comparisons in the broader mCRC population showed OS differences, CRLM-specific conversion trials have not consistently shown that intensifying induction therapy improves OS despite higher resection rates. This suggests that achieving resectability and pursuing curative-intent local treatments in eligible patients remain key determinants of long-term survival and should be the treatment goal.

### 3.2. Liver-Directed Therapies for Conversion and Disease Control

#### 3.2.1. Transarterial Chemoembolization (TACE) and Selective Internal Radiation Therapy (SIRT)

Several liver-directed therapies have been investigated in CRLM, though evidence supporting their routine use remains limited. TACE involves hepatic artery catheterization to deliver chemotherapy combined with arterial embolization, most commonly using irinotecan-loaded drug-eluting beads (DEBIRI). A 2024 Cochrane systematic review of three small RCTs concluded that the evidence for TACE versus systemic chemotherapy is of very low certainty, with uncertain effects on mortality, OS, and PFS [91]. A single-center RCT of 168 patients with chemorefractory unresectable CRLM found that adding TACE to systemic chemotherapy modestly improved PFS (6.7 vs. 3.8 months; HR 0.67; *p* = 0.009) but did not prolong OS (18.4 vs. 14.8 months; *p* = 0.669) [92].

SIRT (Y-90 radioembolization) delivers beta-emitting yttrium-90 microspheres via the hepatic artery. The combined analysis of the FOXFIRE, SIRFLOX, and FOXFIRE-Global phase III trials (n = 1103) demonstrated that adding SIRT to first-line FOLFOX improved objective response rates (72% vs. 63%; *p* = 0.001) and hepatic disease control but did not improve PFS or OS [93]. Notably, first progression in the liver was reduced (cumulative 12-month hepatic progression: 22% vs. 39%), but extrahepatic progression was higher in the SIRT arm (33% vs. 19% at 12 months) [93]. The EPOCH trial, evaluating SIRT in the second-line setting, similarly showed improved PFS (HR 0.69) and hepatic PFS (HR 0.59) without an OS benefit (HR 1.07), with more frequent adverse events [94]. The ASCO guideline does not recommend routine use of SIRT (weak recommendation based on low-quality evidence), citing the absence of OS benefit and increased toxicity [95]. However, SIRT retains a role in selected patients with chemorefractory liver-dominant disease and as a radiation lobectomy approach for FLR augmentation in borderline resectable cases, though evidence remains limited [96,97].

#### 3.2.2. Role of Hepatic Arterial Infusion (HAI)

Hepatic arterial infusion (HAI) delivers high-dose regional chemotherapy, most commonly floxuridine (FUDR), directly to liver metastases via the hepatic artery. When added to modern systemic therapy, HAI has demonstrated meaningful conversion rates and survival improvements in both pretreated and chemotherapy-naïve patients. In a retrospective case–control study of patients with extensive hepatic tumor burden who had received ≥6 months of prior systemic therapy, the addition of HAI-FUDR more than doubled median OS compared with systemic therapy alone (32.8 vs. 15.3 months), with a 12% conversion to resection rate [98]. The OPTILIV trial, a prospective multicenter phase II study of patients who had failed one to three prior systemic regimens, achieved a 29.7% macroscopic complete resection rate with HAI combined with systemic chemotherapy [99]. In a prospective phase II trial at Memorial Sloan Kettering Cancer Center, HAI-FUDR plus systemic chemotherapy achieved a 52% conversion rate by 5 months, with significantly better outcomes in chemotherapy-naïve patients (response rates 86% vs. 67%; 5-year OS 51.9% vs. 27.9%) [100].

Despite strong conversion rates and durable outcomes in expert hands, HAI remains concentrated in high-volume programs with dedicated teams [100]. Feasibility studies have shown that pump implantation and early delivery are achievable with acceptable morbidity, paving the way for multicenter trials [101]. Randomized studies now in progress will test whether the benefits observed in single-center and prospective cohorts translate in a trial setting [11,102].

#### 3.2.3. Stereotactic Body Radiotherapy

SBRT delivers ablative doses of radiation in a limited number of fractions, offering a non-invasive alternative for patients with unresectable CRLM or those in whom thermal ablation is not feasible. A systematic review of 18 studies (656 patients) reported pooled 1- and 2-year OS of 67% and 57%, and 1- and 2-year local control of 67% and 59%, respectively [103]. A moderate correlation between biologically effective dose (BED) and local control was observed (R = 0.47), supporting dose escalation for durable disease control [103]. A multi-institutional Dutch–Belgian registry of 515 patients (80% CRC primary) confirmed 1-year local control of 87% and 1-year OS of 84%, with grade ≥ 3 toxicity in only 3.9% [104]. More recent single-center data suggest that higher BED thresholds (≥137.7 Gy) further improve local control (HR 0.25) and that RAS mutations and larger tumor size are adverse prognostic factors for OS [105].

Comparative data suggest SBRT may offer superior local control over thermal ablation for tumors ≥ 2 cm when compared with radiofrequency ablation (RFA) and >3 cm when compared with microwave ablation (Section 3.3.1). In a propensity-weighted single-institution comparison of SBRT versus RFA for intrahepatic metastases, SBRT was associated with improved freedom from local progression for tumors ≥ 2 cm (HR 0.28; *p* < 0.01), while outcomes were similar for smaller lesions [106]. A meta-analysis of liver metastases studies confirmed higher 2-year local control with SBRT versus RFA (83.6% vs. 60.0%; *p* < 0.001) [107].

### 3.3. After Conversion: Response Evaluation, Resectability, and Local Control

Accurate assessment of treatment response is essential for surgical planning after conversion chemotherapy. Morphologic response criteria are primarily based on the Response Evaluation Criteria in Solid Tumors (RECIST), which primarily focuses on size reduction [108]. Morphologic criteria incorporating changes in lesion attenuation, border definition, and internal homogeneity have been shown to outperform RECIST in predicting pathologic response [109].

A key challenge is the “disappearing liver metastasis”—lesions that become radiographically undetectable after chemotherapy. Computed tomography (CT) has a positive predictive value (PPV) of only 35% for true complete pathologic response at these sites, and persistent viable tumor or early in situ recurrence is found at 83% of disappeared lesion sites [110,111]. Conversely, Gd-EOB-DTPA-enhanced magnetic resonance imaging (MRI) has superior diagnostic performance in evaluating disappearing CRLM, with a 78% PPV for true absence of tumor, and should be the preferred modality when evaluating post-chemotherapy response, particularly for subcentimeter or deep parenchymal lesions [110,112]. The prospective DREAM study, however, showed that even when lesions had disappeared on both CT and MRI, the negative predictive value for the absence of viable tumor was only 62.5% [113]. Intraoperative adjuncts, including IOUS and ICG fluorescence imaging, may help localize residual disease at these sites (Section 2.3.3).

Technical resectability should be reassessed by the MDT after 6–8 weeks of induction chemotherapy and at the same interval thereafter, with surgery proceeding as soon as all original disease sites become amenable to complete local treatment [78]. The prospective RAXO study demonstrated the value of this strategy by noting a 34% discrepancy rate between expert panel evaluation of resectability and local judgement, while highlighting that repeated assessment identified an additional 21% of patients for liver resection, ultimately achieving a 5-yr OS of 65% (R0/R1), vs. 39% (R2/ablation) vs. 2% (non-resected) [114].

These findings support resection or ablation of all original metastatic sites whenever technically feasible, even when lesions are no longer visible on imaging, and using hepatobiliary-phase MRI rather than CT alone to guide these decisions. Once resectability is confirmed, surgery should proceed without further delay after an interval of at least 4 weeks from the last chemotherapy cycle and at least 6 weeks after bevacizumab [17].

#### 3.3.1. Combined Resection and Ablation

When complete clearance by resection alone would require excessive parenchymal sacrifice, combining hepatectomy with thermal ablation offers a parenchyma-sparing alternative. In patients with ≥4 CRLM, resection plus ablation yielded OS and DFS comparable to resection alone (median OS 36 months in both groups), supporting the oncologic adequacy of this approach [115].

Ablation performance is size- and location-dependent. In a per-lesion analysis of 416 tumors (90% treated concomitantly with resection), the 24-month local recurrence rate was 10.9% overall but only 6.8% for tumors ≤ 10 mm, with progressively higher failure beyond 20 mm; subcapsular location independently increased recurrence risk [116]. When thermal margins are constrained by tumor size (>3 cm) or proximity to critical structures, SBRT is an alternative (as discussed in Section 3.2.3). A propensity-weighted comparison of SBRT versus microwave ablation (MWA) for liver metastases ≤4 cm showed that SBRT achieved longer freedom from local progression, with the advantage concentrated in tumors > 30 mm (adjusted HR 0.31) [117].

These data support a multimodal local control strategy combining resection for the dominant disease, MWA for small intraparenchymal lesions where adequate margins are achievable, and SBRT where thermal ablation is technically disadvantaged.

#### 3.3.2. Re-Resection of Recurrence

Despite technically successful resection, recurrence develops in up to 70% of patients; the liver is the most common site of failure, with intrahepatic recurrence occurring in approximately 40–50% of all resected patients, most commonly within the first two years [118,119]. Repeat hepatectomy is feasible in a substantial proportion of these patients and is associated with improved survival compared with systemic therapy alone [120]. In a propensity-matched analysis, repeat hepatectomy yielded a 5-year post-recurrence OS of 19% versus 3% (*p* = 0.048) with systemic therapy alone [120]. This finding was also observed in a meta-analysis of 27 studies (7226 patients) showing that repeat resection is independently associated with improved OS in well-selected patients (HR 0.68, *p* = 0.022) [121]. For recurrences not amenable to re-resection, thermal ablation or SBRT can provide local control, applying the same size- and location-based selection criteria outlined in Section 3.3.1.

#### 3.3.3. Adjuvant HAI

Given that intrahepatic recurrence accounts for most relapses after hepatectomy, adjuvant HAI combined with systemic chemotherapy has been investigated as a strategy to mitigate hepatic recurrence. A large multicenter study found that adding HAI to systemic therapy reduced the five-year cumulative incidence of initial intrahepatic recurrence from 38.4% to 22.9% and improved both DFS and OS [122]. Adjuvant HAI after repeat resection or ablation nearly doubled hepatic DFS (median 46 vs. 19 months) and improved median OS (92 vs. 57 months) [123]. Randomized data, however, remain limited. The HARVEST trial was stopped early for drug supply issues and did not meet its primary endpoint of 3-year recurrence-free survival (RFS), though 1-year RFS favored the HAI arm with acceptable hepatic toxicity [124]. The PACHA-01 trial provides the first positive randomized evidence, demonstrating that adjuvant HAI oxaliplatin plus systemic LV5FU2 significantly improved hepatic RFS (median 25 vs. 12 months; HR 0.63, *p* = 0.047) and overall RFS (median 14 vs. 9 months; HR 0.63, *p* = 0.03) in patients with ≥4 resected CRLM, with a favorable but non-significant OS trend (5-year OS 62% vs. 47%; HR 0.61, *p* = 0.11) [125]. Notably, this trial used oxaliplatin via percutaneous catheter rather than the surgically implanted pump with floxuridine used in North American series.

Overall, at experienced centers, HAI may be integrated as an adjuvant option after curative-intent local therapy to lower early hepatic relapse and improve long-term outcomes, although ongoing trials will further define this role.

## 4. Management of Never Resectable Disease

Never resectable CRLM encompasses patients with liver-only disease that cannot be completely cleared despite advanced surgical techniques, and those with unresectable extrahepatic disease reflecting aggressive biology [126]. In this review, “never resectable” denotes disease that cannot be rendered amenable to complete (R0/R1) local treatment by any available strategy; incomplete (R2) or debulking resection may still be performed in selected patients, as discussed in Section 4.2. This section focuses on patients with liver-only, never resectable disease, in whom technical factors, rather than systemic disease burden, define unresectability as determined through multidisciplinary assessment [9,126]. Patients with unresectable extrahepatic disease are generally managed with systemic therapy and are not candidates for liver transplantation, as transplant protocols, including TransMet, have been restricted to liver-only disease. Historically, the only option for patients with never resectable CRLM was palliative chemotherapy, with a poor prognosis (1- and 5-year OS of 58.1% and 2.2%, respectively, in a population-based cohort of patients receiving palliative chemotherapy) [127]. More recently, liver transplantation and surgical debulking have been studied as management options with the aim of improving local disease control and extending survival [127,128,129].

### 4.1. Liver Transplantation for CRLM

Liver transplantation has emerged as a potential treatment option for carefully selected patients with unresectable CRLM. While traditionally considered a contraindication due to poor outcomes in early studies, advancements in imaging, surgical techniques, oncological therapies, and patient selection criteria have led to renewed interest in this approach [130].

The SECA-I study reported a 5-year OS rate of 60%, with improved results among patients with low clinical risk [131]. Refinement of the selection criteria in SECA-II included a documented chemotherapy response, controlled tumor burden, and a minimum observation period, which led to 5-year OS estimates of 83% [132]. The TransMet RCT demonstrated a significant survival advantage for transplantation, achieving 5-year OS of 56.6% in liver transplantation plus chemotherapy compared with 12.6% in those treated with chemotherapy alone (HR 0.37, *p* = 0.0003), with the per-protocol survival approaching 73% [9]. Recurrence after transplantation is common but is predominantly pulmonary, indolent, and often amenable to treatment, which is distinctly different from post-resection recurrence [9].

However, real-world data suggest outcomes worsen as the indication expands beyond highly selected trial populations. A UNOS registry analysis of 158 patients across 33 U.S. centers reported 3-year OS of 66.7% and DFS of 44.0%, with significantly better outcomes after living-donor versus deceased-donor transplantation (3-year OS 74.1% vs. 57.3%, *p* = 0.04) [133]. A pooled meta-analysis of 23 studies found a 5-year OS of 49.4% with a recurrence rate of 63.5%, highlighting substantial heterogeneity as selection criteria vary across centers [134].

The gap between trial and real-world results is driven primarily by heterogeneity in selection criteria, center experience, and donor type rather than the fundamental failure of the transplant concept itself [134]. These findings underscore that patient selection remains the defining determinant of success, with the best outcomes achieved when eligibility integrates clinical risk scores, metabolic tumor volume, and objective response to chemotherapy within a standard framework [135].

### 4.2. Debulking for CRLM

Although debulking is not traditionally part of the CRLM treatment paradigm, emerging data suggest that selected patients with never resectable liver metastases may derive meaningful benefit from tumor volume reduction. Analyses from the LiverMetSurvey reported notable long-term survival among patients with R2 resections, which was substantially better than outcomes expected with chemotherapy alone [136]. The randomized phase II EORTC CLOCC trial, although not powered for a comparative OS analysis, showed longer survival on long-term follow-up when thermal ablation was added to chemotherapy in patients with unresectable liver-only disease, supporting the potential value of reducing intrahepatic tumor burden even when complete resection is not feasible [137]. Mazzotta et al. compared patients undergoing curative-intent hepatectomy with those undergoing debulking hepatectomy and found no significant difference in OS (3-year OS: 69% vs. 64%; 5-year OS: 47% vs. 35%; *p* = 0.14), though PFS was significantly shorter in the debulking group (*p* = 0.009) [10]. Similarly, a retrospective cohort study found that patients who underwent any form of debulking—whether resection of all visible disease or first stage of an incomplete two-stage hepatectomy—achieved a median OS of 31 months compared with 19.5 months in patients who were never resected (*p* = 0.02) [138].

The ORCHESTRA trial (NCT01792934) is the first and only completed randomized phase 3 trial of debulking in multiorgan mCRC and showed no OS benefit compared to chemotherapy alone (30 vs. 27.5 months; HR 0.88, *p* = 0.26) [139]. However, the inclusion of patients with widespread multiorgan disease limits applicability to liver-dominant settings where debulking strategies may be more biologically relevant.

The role for debulking remains unclear and the theoretical therapeutic gain must be weighed against operative morbidity and delays in systemic therapy. Despite these uncertainties, several arguments support continued investigation. Preclinical and translational data suggest tumor volume reduction may enhance systemic therapy efficacy by eliminating chemoresistant clones and restoring antitumor immunity; retrospective analyses suggest survival outcomes that exceed those achieved with chemotherapy alone, and studies including locoregional therapies have demonstrated the value of controlling intrahepatic disease when complete resection is not possible [136,137,140,141]. These considerations support evaluating debulking hepatectomy in patients with liver-dominant unresectable disease within a prospective clinical trial that applies strict selection criteria to determine which patients can meaningfully benefit.

### 4.3. Histotripsy

Histotripsy is a non-invasive, non-thermal focused ultrasound technology that mechanically disrupts tissue through controlled acoustic cavitation [142,143]. Unlike thermal ablation, histotripsy does not generate heat and therefore avoids the heat-sink effect near major vessels [144]. To date, clinical evidence is limited to early-phase studies conducted predominantly in patients with advanced disease who failed or were ineligible for surgery, thermal ablation, and systemic therapy [145,146]. Early clinical data from the THERESA trial and the #HOPE4LIVER study demonstrated technical success rates exceeding 95% with a favorable safety profile [146,147]. A recent systematic review and meta-analysis including 553 patients confirmed technical feasibility and safety but emphasized that long-term oncologic outcomes, comparative effectiveness data, and standardized response assessment criteria are lacking [148]. While histotripsy may represent a promising addition to the liver-directed therapy armamentarium, its role in CRLM management remains investigational, and long-term efficacy data are needed before it can be considered an alternative to established techniques.

## 5. Discussion and Future Directions

**Figure 3:**
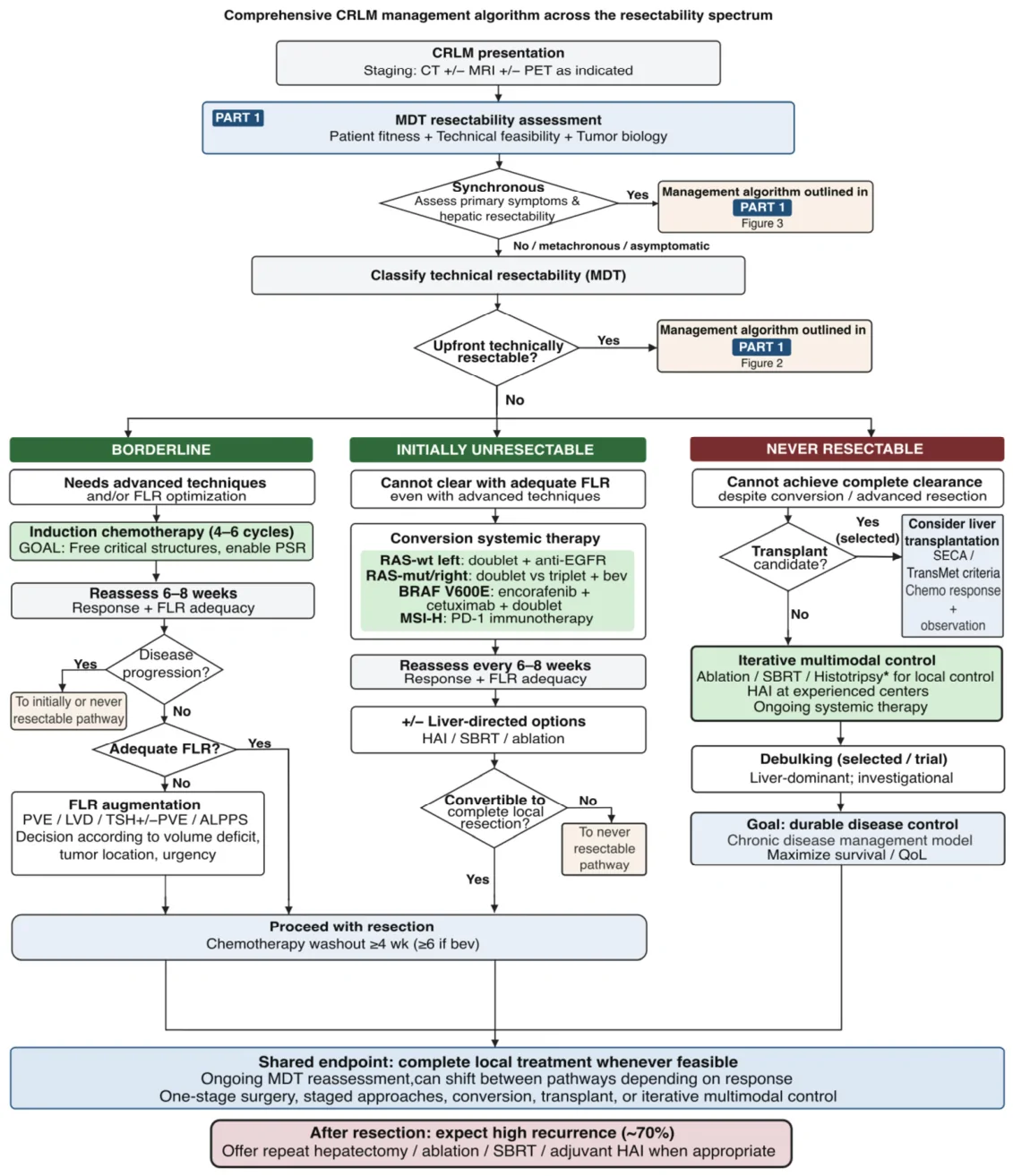
Proposed multidisciplinary management algorithm for CRLM across the resectability spectrum. Initial assessment of resectability comprises patient, biologic, and technical factors, with repeated multidisciplinary evaluation as treatment response and disease biology evolve. Management strategies incorporate systemic therapy, liver-directed therapies, FLR optimization, staged resection, transplantation in select patients, and iterative multimodal control. Arrows indicate the sequence of assessment and management within the proposed treatment algorithm. Management algorithms for synchronous disease and upfront technically resectable CRLM are presented in Part 1 of this review. * Histotripsy is currently trial-only. CRLM, colorectal liver metastases; CT, computed tomography; MRI, magnetic resonance imaging; PET, positron emission tomography; MDT, multidisciplinary team; FLR, future liver remnant; PSR, parenchymal-sparing resection; RAS-wt, RAS wild-type; EGFR, epidermal growth factor receptor; RAS-mut, RAS-mutant; bev, bevacizumab; MSI-H, microsatellite instability-high; PD-1, programmed cell death protein 1; HAI, hepatic arterial infusion; SBRT, stereotactic body radiotherapy; PVE, portal vein embolization; LVD, liver venous deprivation; TSH, two-stage hepatectomy; ALPPS, associating liver partition and portal vein ligation for staged hepatectomy; SECA, Secondary Cancer study; QoL, quality of life.

While Part 1 of this review established the principles governing resectable and synchronous CRLM, the management of borderline resectable, initially unresectable, and never-resectable disease introduces a fundamentally different set of challenges (Figure 3). In these settings, treatment decisions are shaped by the interplay between systemic therapy efficacy and patient selection, yet the evidence base is constrained by heterogeneous trial populations, inconsistent definitions of resectability, and a paucity of CRLM-specific randomized studies. A central theme that emerges throughout this review is the tension between the pursuit of complete disease clearance as the only acceptable curative endpoint and the growing recognition that long-term survival may instead depend on the ability to achieve and maintain local disease control through sequential, multimodal local therapy.

### 5.1. The Evidence Gap

The systemic therapy regimens used for conversion in CRLM are largely derived from trials designed for the broader mCRC population. The FIRE-3, CALGB/SWOG 80405, TRIBE, and PARADIGM trials enrolled heterogeneous populations with varying metastatic disease distribution, and conversion to resection was typically a secondary or exploratory endpoint [7,8,66,71]. The CAIRO5 and OLIVIA trials represent notable exceptions, both prospectively evaluating systemic regimens specifically in initially unresectable CRLM [59,78]. The OLIVIA study, restricted to patients with liver-limited disease, showed higher resection rates with FOLFOXIRI plus bevacizumab than with mFOLFOX6 plus bevacizumab (Section 3.1.3), confirming the value of intensified therapy in maximizing conversion in this population [59]. However, the CAIRO5 trial, which incorporated centralized resectability assessment, found that although intensified therapy achieved higher conversion rates, this did not translate into an OS difference. Rather, patients who achieved complete local treatment had markedly improved survival regardless of regimen [6]. These trials carry the implication that regimen intensity may increase the likelihood of achieving resection, but the ability to achieve complete local treatment, rather than the specific systemic regimen used, is the primary determinant of long-term outcome.

### 5.2. Defining Resectability: A Moving Target

Compounding the evidence gap is the absence of a universally accepted definition of resectability. What one center deems unresectable may be considered borderline or even resectable at a high-volume hepatobiliary center with expertise in PVE/HVE, two-stage hepatectomy, or combined resection-ablation strategies. This heterogeneity in classification directly affects reported conversion rates, resection outcomes, and the conclusions drawn from clinical trials. Furthermore, the persistent dissociation between volumetric hypertrophy and functional recovery after rapid augmentation techniques suggests that current volume-based thresholds alone may be insufficient for operative planning, and that functional assessment should be integrated into future resectability frameworks [47,48]. Standardized, multidisciplinary resectability assessment, ideally with centralized expert panel review, should be a prerequisite for future CRLM-specific trials.

### 5.3. Expanding the Toolkit

The therapeutic armamentarium for CRLM continues to expand, though evidence supporting individual modalities varies considerably. HAI has demonstrated strong conversion rates in the unresectable setting, with the PACHA-01 trial now providing the first randomized evidence of improved hepatic RFS using oxaliplatin-based HAI via percutaneous catheter, demonstrating that the principle extends beyond a single drug or delivery platform [125]. By contrast, the evidence for TACE and SIRT/Y-90 remains limited, with randomized trials failing to demonstrate an OS benefit when added to first-line chemotherapy [91,92,93]. SBRT has emerged as a useful liver-directed modality, particularly for lesions where thermal ablation margins are constrained by size or proximity to critical structures, and complements MWA in enabling combined resection-ablation strategies that preserve parenchyma without compromising oncologic outcomes.

### 5.4. Reframing Never Resectable Disease: From Cure or Palliation to Chronic Disease Management

This evolution raises a broader conceptual question. Traditionally, CRLM has been viewed through a binary framework in which patients either undergo complete (R0) resection with curative intent or receive palliative systemic therapy. Increasingly, however, this distinction appears overly simplistic. Modern management frequently relies on staged interventions, repeat hepatectomy, ablation, regional therapies, and systemic treatment delivered over the course of years. Likewise, as outlined in Section 3.3.2, recurrence after hepatectomy is common and most often intrahepatic, and iterative resection or ablation has become standard practice, reflecting an implicit recognition that complete local treatment is often achieved through a sequence of interventions rather than a single operation [118,119].

Perhaps the most consequential unresolved question is whether this evolving paradigm should extend to carefully selected patients traditionally classified as “never resectable.” Current guidelines discourage partial cytoreduction, or debulking, a position reinforced by the ORCHESTRA trial, which found no survival benefit from adding tumor debulking to first-line palliative chemotherapy in patients with multiorgan mCRC [18,139]. However, the trial enrolled patients with widespread multiorgan disease, used doublet chemotherapy without anti-EGFR agents, and was not designed to evaluate debulking in patients with liver-only or liver-dominant disease, where the biological rationale for tumor volume reduction is strongest. The EORTC CLOCC trial demonstrated that multimodal local treatment of all visible liver metastases using RFA ± resection when surgical R0 resection alone was not feasible improved OS at nearly 10 years (HR 0.58, *p* = 0.01), providing randomized, albeit phase II, evidence that local disease clearance through combined modalities may extend survival even in patients classified as surgically unresectable [137].

The field may benefit from reconceptualizing liver-dominant CRLM as a potentially chronic disease amenable to iterative multimodal management, combining resection, ablation, SBRT, HAI, and systemic therapy in a coordinated sequence rather than a condition that must be cured or relegated to palliative chemotherapy.

## 6. Conclusions

The management of borderline resectable, initially unresectable, and never resectable CRLM demands individualized, biology-informed decision-making within a multidisciplinary framework. While the therapeutic armamentarium has expanded considerably, the evidence base remains constrained by the absence of CRLM-specific trial designs, inconsistent definitions of resectability, and reliance on retrospective data for many key decisions. The field would benefit from collaborative, multicenter trials that apply standardized resectability criteria, incorporate modern systemic regimens and molecular profiling, and test iterative multimodal strategies in liver-dominant disease.

## Figures and Tables

**Table 1:** Common chemotherapeutic regimens for CRLM.

Regimen	Common Side Effects (Grade 1–2)	Serious Side Effects (Grade 3–4)
**FOLFOX** (5-FU, leucovorin, oxaliplatin)	Peripheral sensory neuropathy (oxaliplatin-related) Neutropenia Fatigue Diarrhea Nausea/vomiting Mucositis Hand–foot syndrome (5-FU–related)	Severe cumulative neurotoxicity (oxaliplatin-related) Hypersensitivity reactions to oxaliplatin
**CAPOX** (capecitabine, oxaliplatin)	Peripheral sensory neuropathy (oxaliplatin-related) Hand–foot syndrome (capecitabine-related) Diarrhea Nausea/vomiting Fatigue	Severe neurotoxicity (oxaliplatin-related) Hypersensitivity reactions to oxaliplatin Increased toxicity risk in patients with renal impairment (capecitabine-related)
**FOLFIRI** (5-FU, leucovorin, irinotecan)	Diarrhea Nausea/vomiting Fatigue Neutropenia Alopecia	Severe neutropenia Severe diarrhea leading to dehydration Increased toxicity in UGT1A1 polymorphisms (irinotecan-related)
**FOLFOXIRI** (5-FU, leucovorin, oxaliplatin, irinotecan)	Diarrhea Nausea/vomiting Fatigue Neutropenia Alopecia Neurotoxicity (oxaliplatin-related)	Severe diarrhea (irinotecan-related). Severe neutropenia Cumulative neurotoxicity (oxaliplatin-related) Increased GI toxicity from combined agents

CRLM, colorectal liver metastases; 5-FU, 5-Fluorouracil; UGT1A1, UDP glucuronosyltransferase 1 family, polypeptide A1; GI, gastrointestinal.

**Table 2:** Trials informing biologic agent selection in initially unresectable mCRC.

Trial	Design/Population	Treatment Arms	Response and Resection Outcomes	Survival Outcomes
FIRE-3 [7]	Phase III RCT RAS-wt mCRC	FOLFIRI + cetuximab vs. FOLFIRI + bevacizumab	ORR: 65.5% vs. 59.6% (*p* = 0.32) Resection: NR	PFS: 10.4 vs. 10.2 mo (HR 0.93, *p* = 0.54); OS: 33.1 vs. 25.6 mo (HR 0.7, *p* = 0.011)
PEAK [69,70]	Phase II RCT RAS-wt mCRC	mFOLFOX6 + panitumumab vs. mFOLFOX6 + bevacizumab	ORR: 65% vs. 60% (*p* = 0.86) Resection: NR	PFS: 13.0 vs. 9.5 mo (HR 0.65, *p* = 0.029); OS: 41.3 vs. 28.9 mo (HR 0.63, *p* = 0.058)
PARADIGM [8]	Phase III RCT RAS-wt mCRC	mFOLFOX6 + panitumumab vs. mFOLFOX6 + bevacizumab	Left-sided ORR: 80.2% vs. 68.6% R0 resection: 18.3% vs. 11.6% (left), 16.5% vs. 10.9% (all)	Left-sided OS: 37.9 vs. 34.3 mo (HR 0.82, *p* = 0.03) All—OS: 36.2 vs. 31.3 mo (HR 0.84, *p* = 0.03)
CALGB/SWOG 80405 [71]	Phase III RCT KRAS-wt mCRC	FOLFOX or FOLFIRI + cetuximab vs. FOLFOX or FOLFIRI + bevacizumab	ORR: 59.6% vs. 55.2% (*p* = 0.13) Resection: NR	PFS: 10.5 vs. 10.6 mo (HR 0.95, *p* = 0.45) OS: 30 vs. 29 mo (HR 0.88, *p* = 0.08)
CELIM [72]	Phase II, two-arm KRAS-wt unresectable CRLM	FOLFOX + cetuximab vs. FOLFIRI + cetuximab	ORR: 68% vs. 57% (*p* = 0.23) Resection: 60% in KRAS-wt patients	PFS: NR OS: 35.7 mo pooled
VOLFI [73]	Phase II RCT RAS-wt mCRC	FOLFOXIRI + panitumumab vs. FOLFOXIRI	ORR: 87.3% vs. 60.6% (*p* = 0.004) Resection: 33.3% vs. 12.1% (*p* = 0.02)	PFS: 9.7 vs. 9.7 mo (*p* = 0.76); OS: 35.7 vs. 29.8 mo (*p* = 0.12)
TRIPLETE [74,75]	Phase III RCT RAS/BRAF-wt mCRC	FOLFOXIRI + panitumumab vs. FOLFOX + panitumumab	ORR: 73% vs. 76% R0 resection: 25% vs. 29%	PFS: 12.7 vs. 12.3 mo (HR 0.88, *p* = 0.19); OS: 41.1 vs. 33.3 mo (HR 0.79, *p* = 0.049)
BECOME [76]	Phase II RCT RAS-mut CRLM	mFOLFOX6 + bevacizumab vs. mFOLFOX6	ORR: 54.5% vs. 36.8% (*p* < 0.01) R0 resection: 22.3% vs. 5.8% (*p* = 0.01)	PFS: 9.5 vs. 5.6 mo (HR 0.49, *p* < 0.001); OS: 25.7 vs. 20.5 mo (HR 0.71, *p* = 0.03)
TRIBE [66,77]	Phase III RCT mCRC	FOLFOXIRI + bevacizumab vs. FOLFIRI + bevacizumab	ORR: 65% vs. 53% (*p* = 0.006) R0 resection: 15% vs. 12% (*p* = 0.33)	PFS: 12.1 vs. 9.7 mo (HR 0.75, *p* = 0.003); OS: 29.8 vs. 25.8 mo (HR 0.80, *p* = 0.03)
OLIVIA [59]	Phase II RCT unresectable CRLM	FOLFOXIRI + bevacizumab vs. mFOLFOX6 + bevacizumab	ORR: 81% vs. 62% (*p* = 0.045) R0 resection: 49% vs. 23% (*p* = 0.013)	PFS: 18.6 vs. 11.5 mo (HR 0.43, *p* = 0.005); OS: NR
CAIRO5 [6,78]	Phase III RCT RAS/BRAF-mut or right-sided unresectable CRLM	FOLFOXIRI + bevacizumab vs. FOLFOX/FOLFIRI + bevacizumab	ORR: 54% vs. 43% (*p* = 0.0004) complete local treatment: 51% vs. 36% (*p* = 0.013)	PFS: 10.6 vs. 9 mo (HR 0.76, *p* = 0.032); OS: 24.1 vs. 23.6 mo (HR 0.9, *p* = 0.44
BREAKWATER [79,80]	Phase III RCT BRAF V600E-mut mCRC	Encorafenib + cetuximab + mFOLFOX6 vs. standard of care	ORR: 60.9% vs. 40.0% (*p* < 0.001) Resection: NR	PFS: 12.8 vs. 7.1 mo (HR 0.53, *p* < 0.001); OS: 30.3 vs. 15.1 mo (HR 0.49, *p* < 0.001)
KEYNOTE-177 [81]	Phase III RCT MSI-H/dMMR mCRC	Pembrolizumab vs. chemotherapy +/− bevacizumab/cetuximab	ORR: 45.8% vs. 33.1% Resection: NR	PFS: 16.5 vs. 8.2 mo (HR 0.60, *p* = 0.0002); OS: 77.5 mo vs. 36.7 mo (HR 0.73, 95% CI 0.53–0.99)

RCT, randomized controlled trial; ORR, objective response rate; PFS, progression-free survival; mo, months; mCRC, metastatic colorectal cancer; NR, not reported; HR, hazard ratio; CRLM, colorectal liver metastases; wt, wild-type; mut, mutated; MSI-H, microsatellite instability high; dMMR, deficient mismatch repair.

**Table 3:** Approach to systemic therapy based on resectability *.

Setting	Molecular/Clinical Subset	Preferred Regimen	Key Evidence	Practical Notes
**Borderline resectable**	RAS/BRAF wild-type, or left-sided	Doublet (FOLFOX or FOLFIRI; anti-EGFR not recommended	Inferior OS with perioperative cetuximab in resectable/suboptimally resectable CRLM (New EPOC)	Short induction, reassess at 6–8 weeks ≥4-week washout before surgery
	RAS mutated, or right-sided	Doublet (FOLFOX or FOLFIRI) ± bevacizumab	Bevacizumab favored over anti-EGFR in these subsets (FIRE-3, CALGB/SWOG 80405) ^‡^	Short induction, reassess at 6–8 weeks ≥4-week washout before surgery (>6 weeks after bevacizumab)
**Initially unresectable**	Fit, liver-limited, need maximal shrinkage	Triplet (FOLFOXIRI) + bevacizumab	Higher response rate and PFS than doublet; OS benefit in TRIBE/TRIBE 2 but not in CAIRO5. Increased R0 rates in liver-limited disease (OLIVIA, CAIRO5)	Reserve for fit patients Reassess frequently and move to local therapy when criteria met
	RAS/BRAF wild-type, or left-sided	Doublet + anti-EGFR (FOLFOX or FOLFIRI + cetuximab/panitumumab)	High response rates and conversion to resection rate (FIRE-3, CALGB/SWOG 80405, VOLFI)	Consider when triplet toxicity is a concern
	RAS mutated, or right-sided	Doublet (FOLFOX or FOLFIRI) + bevacizumab; consider triplet + bevacizumab if fit	Triplet improves conversion in adverse biology Right-sided and RAS-mutated respond better to bevacizumab-based strategies (CAIRO5, TRIBE, TRIBE2)	Short induction, reassess at 6–8 weeks; proceed to local therapy when criteria met ≥6-week washout before surgery
	MSI-H/dMMR	PD-1-based immunotherapy	Superior disease control and quality of life vs. chemo with durable response (KEYNOTE-177, CheckMate-142)	If cytoreduction is inadequate, discuss hybrid strategies in MDT Timing to surgery is individualized
**Either**	BRAF V600E mutated	Encorafenib + cetuximab + doublet (FOLFOX or FOLFIRI)	ORR 61% Significant OS and PFS advantage compared to previous SOC (doublet + bevacizumab)	New first-line standard Reassess for resection early given high response rates

* Regimen for upfront resectable disease is detailed in Part 1. ^‡^ Evidence extrapolated from trials designed for unresectable mCRC. The only randomized trial enrolling suboptimally resectable CRLM (New EPOC) demonstrated inferior OS with the addition of cetuximab [19]. EGFR, epidermal growth factor; OS, overall survival; PFS, progression-free survival; MSI-H, microsatellite instability-high; dMMR, mismatch repair deficient; PD-1, programmed cell death protein; MDT, multidisciplinary team; ORR, objective response rate; SOC, standard of care.

## Data Availability

No new data were created or analyzed in this study. Data sharing is not applicable to this article.

## Abbreviations

The following abbreviations are used in this manuscript:

5-FU	5-Fluorouracil
ALPPS	Associating liver partition and portal vein ligation for staged hepatectomy
ASCO	American Society of Clinical Oncology
BED	Biologically effective dose
CALI	Chemotherapy-associated liver injury
CAPOX	Capecitabine and oxaliplatin
CI	Confidence interval
CRC	Colorectal cancer
CRLM	Colorectal liver metastases
CT	Computed tomography
DEBIRI	Drug-eluting bead irinotecan
DFS	Disease-free survival
DH	Degree of hypertrophy
dMMR	Deficient mismatch repair
EGFR	Epidermal growth factor receptor
FLR	Future liver remnant
FOLFIRI	Folinic acid, 5-fluorouracil, and irinotecan
FOLFOX	Folinic acid, 5-fluorouracil, and oxaliplatin
FOLFOXIRI	Folinic acid, 5-fluorouracil, oxaliplatin, and irinotecan
FUDR	Floxuridine
Gd-EOB-DTPA	Gadolinium ethoxybenzyl diethylenetriamine pentaacetic acid
HAI	Hepatic artery infusion
HR	Hazard ratio
HVE	Hepatic vein embolization
LVD	Liver venous deprivation
mCRC	Metastatic colorectal cancer
MDT	Multidisciplinary team
MRI	Magnetic resonance imaging
MSI-H	Microsatellite instability-high
MWA	Microwave ablation
ORR	Objective response rate
OS	Overall survival
PD-1	Programmed cell death protein 1
PFS	Progression-free survival
PHLF	Post-hepatectomy liver failure
POLD1	DNA polymerase delta 1
POLE	DNA polymerase epsilon
PPV	Positive predictive value
PVE	Portal vein embolization
RCT	Randomized controlled trial
RECIST	Response Evaluation Criteria in Solid Tumors
RFA	Radiofrequency ablation
RFS	Recurrence-free survival
SBRT	Stereotactic body radiotherapy
SIRT	Selective internal radiation therapy
TACE	Transarterial chemoembolization
TSH	Two-stage hepatectomy
UNOS	United Network for Organ Sharing
VEGF	Vascular endothelial growth factor
Y-90	Yttrium-90
